# Bariatric surgery implementation trends in the USA from 2002 to 2012

**DOI:** 10.1186/s13012-016-0382-x

**Published:** 2016-02-20

**Authors:** Emily E. Johnson, Annie N. Simpson, Jillian B. Harvey, Kit N. Simpson

**Affiliations:** 1 College of Nursing, Medical University of South Carolina, Room 414, 99 Jonathan Lucas Street, Charleston, SC 29425 USA; 2 Department of Health Leadership and Management, Medical University of South Carolina, 151 Rutledge Avenue, Charleston, SC 29425 USA

**Keywords:** Diffusion of innovation, Implementation, Bariatric surgery, Conceptual model

## Abstract

**Background:**

Many beneficial health care interventions are either not put into practice or fail to diffuse over time due to complex contextual factors that affect implementation and diffusion. Bariatric surgery is an example of an effective intervention that recently experienced a plateau and decrease in rates, with minimal documented justification for this trend. While there are conceptual models that provide frameworks of general innovation implementation and diffusion, few studies have tested these models with data to measure the relative effects of factors that affect diffusion of specific health care interventions.

**Methods:**

A literature review identified factors associated with implementation and diffusion of health care innovations. These factors were utilized to construct a conceptual model of diffusion to explain changes in bariatric surgery over time. Six data sources were used to construct measures of the study population and factors in the model that may affect diffusion of surgery. The population included obese and morbidly obese patients from 2002 to 2012 who had bariatric surgery in 15 states. Multivariable models were used to identify environmental, population, and medical practice factors that facilitated or impeded diffusion of bariatric surgery over time.

**Results:**

It was found that while bariatric surgery rates increased over time, the speed of growth in surgeries, or diffusion, slowed. Higher cumulative number of surgeries and higher proportion of the state population in age group 50–59 slowed surgery growth, but presence of Medicare centers of excellence increased the speed of surgery diffusion. Over time, the factors affecting the diffusion of bariatric surgery fluctuated, indicating that diffusion is affected by temporal and cumulative effects.

**Conclusions:**

The primary driver of diffusion of bariatric surgery was the extent of centers of excellence presence in a state. Higher cumulative surgery rates and higher proportions of older populations in a state slowed diffusion. Surprisingly, measures of the presence of champions were not significant, perhaps because these are difficult to measure in the aggregate. Our results generally support the conceptual model of diffusion developed from the literature, which may be useful for examining other innovations, as well as for designing interventions to support rapid diffusion of innovations to improve health outcomes and quality of care.

## Background

Modern health care has been described as encompassing a “quality chasm,” which occurs when beneficial therapies are attained that can improve quality of patient care, yet they are not put into practice or fail to diffuse over time [1, 2]. It has been found that patients may only receive an average 55 % of recommended care, with only moderate variation among gender, age, and race subgroups. Women, patients under the age of 31, blacks, and Hispanics receive slightly less recommended care than men, patients over age of 64, and whites. However, gaps in quality of care between socio-demographic subgroups are small, compared to the overall gap in quality care that the entire population fails to receive [3]. Since there is a significant gap between what medical care should deliver and the care that patients actually receive, research is needed on how to translate experimental research findings into clinical and public health practice [4–6].

### Implementation and diffusion theoretical models

While implementation science identifies factors associated with adoption of health care interventions, a subset of implementation science, diffusion of innovation (DOI) research, focuses on factors that “increase or decrease the likelihood that a new idea, product, or practice” will be spread over time [7]. There have been numerous studies that have created theoretical frameworks and models to identify predictive factors that explain general implementation and diffusion of health care interventions [8–13]. These frameworks encompass classifications of factors identified to be important to implementation and diffusion over time including organizational, adopting user, innovation, external community, and patient characteristics [8–13]. However, few studies have applied data to these theoretical models to identify factors associated with implementation and diffusion of specific interventions.

### Diffusion of bariatric surgery

Bariatric surgery is an effective and cost-effective intervention for obesity and morbid obesity [14–16] that experienced a period of rapid growth, yet recently endured a plateau in rates, with minimal justification for this trend. While bariatric surgery rates increased significantly from 2002 to 2008 [17–19], rates were found to plateau from 2009 to 2012 [17–19]. Of interest to note is that during the time period of 2002–2012, average state-wide obesity and diabetes rates steadily increased from 30.7 to 39.2 % and from 8.0 to 12.7 %, respectively [19], so although obesity and diabetes rates continuously increased, bariatric surgery rates did not correspondingly increase.

There are two published studies that have examined factors that affect adoption of bariatric surgery in US hospitals. A previous study utilized the Agency for Healthcare Research and Quality (AHRQ) data from 1995 to 2000 to determine factors associated with adoption of bariatric surgery at the hospital level in 11 states. This study found that hospitals that adopted bariatric surgery were typically larger for-profit entities that had a higher degree of dependence on managed care, as well as a location in markets, where more hospitals had already adopted the surgery. Interestingly, the state-level obesity rate did not have an effect on adoption of bariatric surgery [20]. Another prior study utilized data from 1971 to 1981 to determine factors associated with diffusion of five common types of surgery, including morbid obesity surgery. Although this study utilized data that is over 30 years old, it found that hospitals with a higher percentage of commercial insurance coverage and areas with higher per capita income and higher percent of whites, as well as more surgical specialists and hospitals with larger bed sizes and teaching hospitals, were more likely to implement new types of surgery while public hospitals were less likely. Surgeon age and board certification were probable associations with adoption of surgery [21].

Both of these previous studies offer a framework of factors to consider when exploring the diffusion of bariatric surgery, while providing opportunity for future research. These studies were completed prior to the period of surgery expansion and plateau and did not include some potential factors identified to be important in DOI, such as regulatory and legislation factors and physician champion saturation [1, 13].

Obesity is a widespread and serious issue, as over 35 % of the US population is obese and obesity rates are predicted to continue to increase along with obesity-related secondary conditions and costs [22, 23]. Although bariatric surgery has been proven to be effective in reducing obesity, this procedure experienced a recent time period of plateau in rates and there is a scarcity of current literature regarding factors related to diffusion of bariatric surgery. The goal of the current study was to create a model to determine which environmental, population, and medical practice factors affect speed of diffusion of bariatric surgery at the state level.

## Methods

### Design of the study

**Fig. 1:**
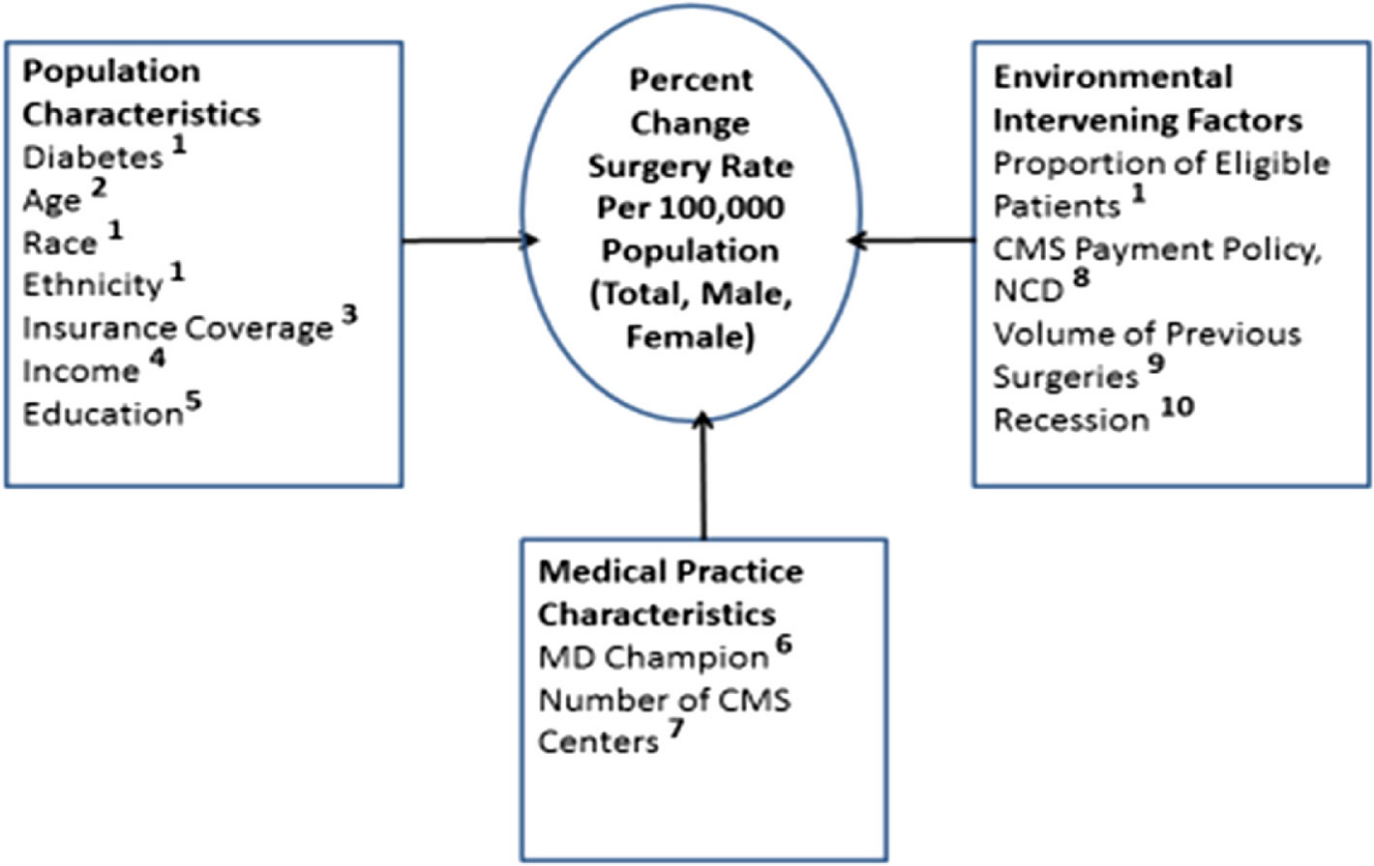
Conceptual model of surgical diffusion. Legend: *1* expressed as state-wide percentage, *2* expressed as state-wide percentage of 10-year age groups, *3* expressed as percent of surgical patients with private insurance, *4* expressed as household median income, *5* expressed as percent of population with high school degree or lower, *6* expressed as number of new publications per 1,000,000 population, *7* expressed as total number of CMS centers per 1,000,000 population, *8* expressed as percent change of surgeries prior to and after 2006, *9* expressed as cumulative previous rate of surgeries per 100,000, *10* expressed as year of economic recession

This was a retrospective study that identified explanatory factors associated with differences in bariatric surgery diffusion rates over time. A full literature review was completed to identify contextual factors associated with diffusion of health care innovations, and these factors were aggregated to form a conceptual model to explain changes in bariatric surgery rates over time (Fig. 1). The model for this study focused on variables within three categories of factors: environmental, population, and medical practice (Fig. 1).

### Overview of data sources and variables

Approval from the Institutional Review Board from the Medical University of South Carolina was obtained. Data from six data sources were used in this study; Agency for Healthcare Research and Quality (AHRQ) Healthcare Cost and Utilization Project (HCUP) State Inpatient Database (SID) [24], Behavioral Risk Factor Surveillance System (BRFSS) [25], United States Census Bureau [26], Bureau of Labor Statistics [27], Centers for Medicare and Medicaid Services (CMS) [28], and a PubMed Systematic Literature Search.

The primary outcome measure was the percent change (PC) of the number of bariatric surgeries per 100,000 population per year, for each state in the study. This was expressed as a continuous value and was calculated by the difference in number of surgeries per 100,000 population from 1 year to the next, divided by number of surgeries per 100,000 population in the prior year, all multiplied by 100. Bariatric surgery percent change calculations were assessed for the entire population ages 30–69 as well as within a subset of males and females ages 30–69 separately.

Outcome variable calculation:

$$ P{C}_{TX}=\left(\left( Bar\_{T}_{yrx}- Bar\_{T}_{yrx-1}\right)/ Bar\_{T}_{yrx-1}\right)\times 100 $$

where PC_Tx_ = total change rate for time (*x*) and Bar_T = rate of bariatric surgery per 100,000 population ages 30–69.

Independent variables in the study are listed in Table 1. These include factors in the categories of environmental, population, and medical practice characteristics that were analyzed to determine an association with diffusion of bariatric surgery.

**Table 1:** Independent variables in study

Variable name	Definition	Source
Environmental variables		
Proportion of eligible patients	State-wide percent obesity	BRFSS [25]
CMS payment policy, NCD	Year prior to National Coverage Decision (NCD) or year post-NCD, a key regulatory legislation of 2006 that limited the eligibility of Medicare patients to specific types of bariatric surgery performed in certified centers of excellence [29]	CMS [29]
Volume of previous surgeries	Cumulative total volume of previous bariatric surgery rates	AHRQ HCUP SID [24]
Recession	Recession was present in 2001 and December 2007–June 2009	Bureau of Labor Statistics [27]
Population characteristics		
Diabetes	State-wide percent diabetes	BRFSS [25]
Age	State-wide percent in age group 30–39, 40–49, 50–59, 60–69	US Census Bureau [26]
Race	State-wide percent black	US Census Bureau [26]
Ethnicity	State-wide percent Hispanic	US Census Bureau [26]
Insurance coverage	State-wide percent private insurance	US Census Bureau [26]
Income	State-wide median family income	US Census Bureau [26]
Education	State-wide percent high school diploma or lower	US Census Bureau [26]
Medical practice characteristics		
MD champion	Number of published peer-review journals on bariatric surgery per state	Pub Med Systematic Literature Review
Number of CMS centers of excellence (COE)	Number of state-wide CMS centers of excellence	CMS [28]

All percentages/counts in the study were standardized with Census Bureau population estimates for each year and state to account for state population differences

### Study population

The study population included all patients age 30 to 69 years old that had bariatric surgery between 2002 and 2012, in a selection of 15 states. This study time period was chosen because of the rapid increase in bariatric surgeries during these years, as well as the passage of the Medicare National Coverage Decision (NCD) that took place in the middle of this time period [29, 30]. The age range of patients was chosen because over this time period, this age range had the highest percentages of bariatric surgeries completed [31]. Utilizing this age range allowed the most eligible and stable study population to be included, based on average ages of bariatric surgery patients and preliminary analyses of patient age demographics [31]. Patients that died during hospitalization were excluded from the study in order to exclude severe trauma, cardiac, and cancer patients that had an ICD9 code for a gastrointestinal procedure that was not bariatric surgery.

The HCUP SID was utilized to identify patients, utilizing ICD-9 procedure codes for bariatric surgery, which include 43.82 (laparoscopic sleeve gastrectomy), 43.89 (open sleeve gastrectomy), 44.31 (high gastric bypass/vertical banded gastroplasty), 44.38 (laparoscopic gastroenterostomy), 44.39 (gastric bypass), 44.68 (laparoscopic gastroplasty/vertical band), and 44.95 (laparoscopic gastric restrictive procedure/LAGB) [32].

The 15 states included in the study include Arizona, California, Colorado, Florida, Iowa, Maryland, Michigan, New Jersey, New York, North Carolina, Rhode Island, South Carolina, Washington, West Virginia, and Wisconsin. These states were chosen because of data availability and diverse geographic representativeness of the population sampled within the USA.

### Statistical analysis

The unit of analysis in this study was the state. Research in implementation science is understudied with no universally accepted research design [33, 34]. Other small area analysis (SAA) and DOI studies have utilized large longitudinal databases, identified standardized utilization rates, and utilized regression for analysis [20, 21, 34, 35]. Due to insufficient clear evidence on best methodology and measurement practices in DOI research, measurement for this study is based on previous SAA and DOI recommendations of standardizing surgery rates by population size and use of regression analysis [35]. Thus, this study utilized simple and multiple regression with bariatric surgery rates standardized to population size in order to determine the environmental, patient, and medical practice characteristic factors that are associated with speed of diffusion of bariatric surgery by state.

To control for average differences over time across states, a fixed effects general linear regression model was constructed. Four separate models were estimated to include effects of explanatory variables on total surgeries by three categories of environmental, patient, and medical practice factors, as well as a parsimonious model that included all significant variables. A fifth model estimated differences in gender relating to diffusion of bariatric surgery. Independent variables were entered individually utilizing simple linear regression, as well as together, utilizing multiple manual backward regression to conclude individual and overall explanatory effects. Non-significant variables (*p* > .05) were removed from the analysis, and the models were re-estimated with significant variables. In addition, population level characteristics (gender) were controlled for in the analysis to determine if this impacts results.

The outcome variable was not normally distributed, which was accounted for with a natural log transformation. Thus, the values of all regression parameter estimates reflect log-transformed percent change. In order to interpret the estimates, they were back transformed. Thus, interpretation of the findings has been transformed back to the original scale, but the parameter estimates (*β*s) are logged. In the analysis, *p* values less than 0.05 were considered significant, and SAS version 9.4 was utilized for data analysis.

## Results

There were 506,313 patients identified in the study population that had bariatric surgery from 2002 to 2012 in 15 states. Seventy-seven percent (391,624) of patients were female. For all states combined, the mean surgery rate per 100,000 population increased from 45.31 in 2002 to 67.95 in 2012. All values for mean total, male, and female bariatric surgery rates per 100,000 population increased over the 10-year period. Mean female rates per 100,000 population were consistently higher than male rates per 100,000 population (Table 2).

**Table 2:** Mean total and percent change bariatric surgery rates per 100,000 population, all states

Year	Total surgeries mean (SD)	Male surgeries mean (SD)	Female surgeries mean (SD)	% Change total surgeries mean (SD)	% Change male surgeries mean (SD)	% Change female surgeries mean (SD)
2002	45.31 (9.03)	17.19 (3.72)	71.96 (14.34)	…	…	…
2003	71.06 (15.03)	28.95 (7.31)	111.76 (23.46)	59.29 (40.19)	72.65 (57.84)	57.50 (37.69)
2004	70.58 (12.88)	28.72 (5.85)	110.98 (20.16)	0.58 (13.06)	0.97 (13.89)	0.63 (13.55)
2005	62.52 (13.70)	27.06 (6.96)	96.65 (20.25)	−10.78 (14.60)	−5.66 (14.03)	−12.04 (15.27)
2006	58.03 (14.50)	26.31 (7.42)	88.56 (21.26)	−7.48 (10.92)	−2.82 (14.11)	−8.59 (10.82)
2007	62.06 (18.30)	28.06 (8.38)	94.70 (27.80)	6.69 (15.11)	7.07 (12.53)	6.62 (16.53)
2008	70.73 (21.60)	33.88 (9.49)	106.35 (33.19)	15.04 (14.39)	22.76 (18.33)	13.10 (13.84)
2009	71.26 (20.74)	36.01 (10.58)	105.12 (30.43)	1.62 (10.61)	6.53 (11.53)	0.009 (11.08)
2010	66.68 (18.22)	33.16 (9.50)	98.85 (26.86)	−5.44 (9.23)	−7.11 (9.69)	−4.96 (9.70)
2011	65.70 (17.69)	31.70 (9.10)	98.35 (26.23)	−1.25 (7.94)	−3.74 (12.14)	−0.36 (7.31)
2012	67.95 (20.19)	32.53 (9.25)	101.78 (30.73)	5.79 (17.61)	7.19 (27.67)	5.58 (15.73)
Grand mean (SD)	64.82 (17.97)	29.47 (9.28)	98.79 (26.95)	6.05 (25.19)	9.37 (31.53)	5.40 (24.57)

Ellipsis signifies rate was not calculated for this year

Conversely, the mean percent change of surgeries per 100,000 population for all states decreased from 59.29 % in 2003 to 5.79 % in 2012, and all mean values of percent change of surgeries per 100,000 population for total, male, and female surgeries for all states decreased over the 10-year period. Diffusion rates were higher among males, but this difference was not significant (Table 2).

There were significant changes in diffusion of surgery over time. Over the 10-year period, time was negatively associated with percent change in bariatric surgery rates for total surgeries (*β* = −0.005, *p* value = 0.001). This indicates that for each additional year in the 10-year time period, percent change of total bariatric surgery decreased by 1.005 % (Table 3).

**Table 3:** Explanatory variables of diffusion of bariatric surgery, by category (*n* = 148)

	Univariate models	Domain models	Final model
	Param. Est. *β* (SE)	Param. Est. *β* (SE)	Param. Est. *β* (SE)
Time			
Intercept	0.0318 (0.0077)*		0.3203 (0.1098)*
Year	−0.0047 (.0015)*		
Environment			
Intercept	NA	0.0532 (0.0296)	
Obesity	−0.0017 (0.0008)*	−0.0006 (0.0009)	
Medicare NCD	−0.0245 (0.0091)		
Cum. surgery rate	−0.00007 (0.00002)*	−0.00006 (0.00002)*	−0.0001 (0.00004)*
Recession year	0.0099 (0.011)		
Population			
Intercept	NA	0.2054 (0.0986)*	
Age group 50–59	−0.0098 (0.0031)*	−0.0088 (0.0032)*	−0.0113 (0.0043)*
Black	0.00029 (0.0005)		
Diabetes	−0.0027 (0.0019)		
High school educ.	−0.00004 (0.0007)		
Hispanic	−0.0005 (0.0005)		
Income	−0.0007 (0.0005)		
Private insurance	0.0009 (0.0004)*	0.0005 (0.0004)	
Medical practice			
Intercept	NA	0.0208 (0.0066)*	
Center of excellence	−0.0058 (0.0029)*	−0.0058 (0.0029)*	0.0185 (0.0063)*
MD champ	−0.0073 (0.0070)		

*R* ^2^ = 0.1376 (final model)

*p < 0.05

### Environmental explanatory variables of diffusion of bariatric surgery

When examining the impact of environmental variables on diffusion of surgery, state-wide obesity rate (*β* = −0.002, *p* < 0.05) and cumulative surgery rate (*β* = *−*0.0007, *p* < 0.05) were significantly negatively associated in univariate analysis, while Medicare legislative and recession indicators were not significantly associated with changes in bariatric surgery (Table 3). When including these factors in a multivariable regression model, cumulative surgery rate (1 % decrease, *β =* −0.0006, *p* < 0.05) had higher explanatory effect than state-wide obesity, as obesity no longer remained significant (Table 3).

### Population explanatory variables of diffusion of bariatric surgery

When examining how population variables were associated with diffusion of bariatric surgery, age group 50–59 years (*β* = − 0.0098, *p* < 0.05) and private insurance (*β* = 0.00009, *p* < 0.05) were significant explanatory variables. Black race, Hispanic ethnicity, diabetes prevalence, educational attainment, and income were not significant. When age group 50–59 and private insurance were included in the same model, only age group 50–59 was significant (1 % decrease, *β* = −0.0088, *p* < 0.05) indicating that age group 50–59 had a larger explanatory effect in surgery diffusion than private insurance (Table 3).

### Medical practice explanatory variables of diffusion of bariatric surgery

When analyzing medical practice variables including numbers of centers of excellence (COE) per 1,000,000 population and number of state physician champions per 1,000,000 population, COE was negatively associated (1 % decrease, *β* = −0.0058, *p* < 0.05) and physician champion was not significantly associated with changes in bariatric surgery (Table 3).

### Relative contributions of explanatory power of three domains

Significant explanatory variables of bariatric surgery diffusion were identified within the three individual domain models (environmental, population, medical practice), yet including the significant variables collectively can illustrate relative importance of each variable in diffusion. In the final parsimonious multivariable model, cumulative surgery rate (1 % decrease, *β =* −0.0001, *p* < 0.05) and 50–59 year age group (1 % decrease, *β* = −0.01, *p* < 0.05) were negatively associated and COE (1 % increase, *β* = 0.02, *p* < 0.05) was positively associated with changes in bariatric surgery. In the final multivariable model, COE was a positive explanatory variable, yet it was negatively associated in the univariate model, signifying that controlling for number of surgeries in the final model, via the cumulative surgery rate, had an effect on COE (Table 3).

### Explanatory variables of diffusion of bariatric surgery: differences by state and gender

There were no significant differences found in percent change of bariatric surgery rates by state or gender, and no significant differences in explanatory variables between states and gender.

## Discussion

The goal of this study was to identify factors affecting diffusion of bariatric surgery over a 10-year period. There has been minimal previous research on diffusion of specific health care interventions, yet this area of study is essential and timely in order to determine how to improve the implementation and diffusion of beneficial health care interventions in medical practice, thereby improving quality of care and health outcomes. This study found that bariatric surgery rates per 100,000 population increased over the 10-year period and reached peak values in 2008–2009. However, percent change of surgeries decreased during the 10-year time frame, signifying that diffusion slowed over time. Females had higher rates of surgery over time, but males had greater diffusion of the procedure. Among the US states, there were no significant differences in diffusion rates over time. This indicates that overall bariatric surgery rate increase and percent change decrease over time can be considered a national trend that is not state specific. There were also no significant differences in factors affecting diffusion of surgery by state or gender, making these factors consistent across geographical area and between males and females.

When examining environmental factors associated with diffusion of bariatric surgery, the dominant variable was cumulative surgery rate, which was negatively associated with the rate of diffusion. It was hypothesized that previous cumulative surgery rate would be positively associated with diffusion of bariatric surgery for a few reasons, including higher rates of advertising, competition, direct-to-consumer marketing, and personal referrals that occur as procedure rates increase in an area. However, since the effect of previous cumulative surgery rate in this study was negative, it may be concluded that there is a saturation point of surgeries, at which additional diffusion will slow or cease. A previous study found that cumulative adoption of bariatric surgery had a positive effect on new adoption of the procedure up to a point. At a point of saturation, hospitals decreased expansion of the intervention [20]. Slowing of diffusion at the point of saturation can result from fewer available patients in an area to seek out and receive the intervention, potential decrease in average population body mass index due to public health programs and policies, lack of availability of additional physicians to perform the surgery, and changes in insurance and payer regulations.

When considering the significance of population variables in explaining diffusion of bariatric surgery, age group 50–59 was the dominant variable and was negatively associated. States with higher percent of the population in the age group 50–59 years had lower rates of diffusion of surgery. Previously, patients age 18–54 accounted for 85 % of all bariatric surgeries while patients 65 years and older accounted for only 1 % of surgeries [31], so there is a distinct higher proportion of surgeries completed in younger age groups. With increasing age, patients undergoing bariatric surgery typically have higher rates of adverse events and higher mortality rates, with a dramatic increase in these negative effects after age 60. In addition, positive weight loss outcomes from bariatric surgery have been shown to be lesser in patients of increasing age. It has been recommended that restriction of bariatric surgery in the older population may be beneficial in order to avoid higher risk and detrimental outcomes from this intervention [36]. While there are not yet official age limitations on the surgery, physicians are aware of the higher risks in the older population and this may limit the diffusion of this intervention in this age group. Also, obese individuals have higher mortality rates and shorter life expectancy so there is a smaller obese population eligible for bariatric surgery as age increases [37].

When examining medical practice variables associated with surgical diffusion, COE was a negative explanatory variable of diffusion of surgery in the univariate model; however, it becomes positively associated with diffusion in the final multivariable model. This variable is related to the CMS NCD of 2006, which limited the eligibility of Medicare patients to specific types of bariatric surgery performed in certified centers of excellence [29]. Previous studies have shown that access to bariatric surgery was more difficult after the NCD of 2006, specifically for minority patients [38, 39]. Since the effect of COE reversed in the univariate and multivariable models, it could be considered a proxy for the number of surgeries. After controlling for the number of surgeries in the final model, via the cumulative surgery variable, as the presence of state-wide COE increased, bariatric surgery increased to a level of saturation, at which point, diffusion slowed or ceased. We cannot tell from our data if the key factor in this relationship was due to lack of surgical capacity, or if the rate of change was due to diminishing demand for the surgery. This finding related to intervention saturation should be the focus of further study.

In the final multivariable model, as the number of COE increased, surgical rates increased potentially due to increased availability of the procedure or physician-induced demand. Physician-induced demand is the concept in which physicians may provide services that patients do not need, and it has been found that areas with higher density of physicians had higher utilization and fees related to surgery [40]. This finding has important policy implications because areas with more physicians may lead to over-utilization of interventions, as influenced by the physicians, while areas with lower density of physicians may not have the resources to offer adequate levels of beneficial interventions. On the policy level, needs assessments can assist in identifying appropriate utilization levels of specific health care interventions. However, increases in COE could also be representative of increased physician champion presence in an area. The physician champion variable in this study was not found to be significant; although, the importance of opinion leaders and champions in the implementation process has been previously documented [13]. Opinion leaders can have a positive or negative impact, depending on their level of respect and the influence that they exert over their colleagues and internal and external environment [13]. There is not a universally accepted way to define and measure physician champion effects on diffusion at the aggregate level, which is also a future research need.

### Limitations

The study population was extracted from an administrative claims database, AHRQ HCUP SID, utilizing ICD-9 diagnosis codes for bariatric surgery. It is possible that there were coding errors in this database and all patients that had bariatric surgery over the 10-year time period of this study were not captured, or patients that did not have bariatric surgery were erroneously included in the study population, as these data were collected for health care reimbursement and not for research. In addition, the BRFSS, utilized for state-wide obesity and diabetes prevalence, is a self-report telephone interview survey, which excludes individuals and households that do not have phones. There is also risk of bias in self-report data since individuals may provide false information [25].

There may also be potential unmeasured differences between the states used in this study due to geographic and cultural differences that could affect diffusion of surgery. However, an effort was made to ensure that the states utilized in the study are representative of different geographical areas of the country as well as a range of population sizes and established state-wide obesity rates. Lastly, because the coefficient of determination (R-squared) value of the final statistical model was relatively low (*R* ^2^ = 0.14), this model cannot be considered to be inclusive of all predictive factors that are associated with diffusion of bariatric surgery. Since unidentified factors associated with diffusion of bariatric surgery still exist, this research model is exploratory and future research could focus on identifying additional patient, physician, environmental, or medical practice factors associated with diffusion of surgery.

## Conclusions

This study illustrated that although there were more bariatric surgeries completed as time progressed, diffusion rates slowed over the 10-year period, which can be contributed to a few identified explanatory factors. The primary driver to diffusion of bariatric surgery was the presence of COE, while the two main barriers to diffusion were cumulative surgery rate and state-wide higher age population proportion.

Although the data in this study support only a few explanatory factors in the posed theoretical model of diffusion, there are some important conclusions that can be made. DOI is not constant but is instead affected by temporal factors that can change over time. From the findings of this study, these factors can relate to physician presence, demand for services, the saturation effect, and composition of population. Similarly, the diffusion of other health care interventions can be affected by varied fluctuating factors. The methods and findings from this research can be utilized by clinicians, policy makers, and researchers in order to determine additional transforming factors associated with other health care innovations in order to increase diffusion of beneficial health care innovations over time, thereby improving health care quality and outcomes and decreasing health care costs in the future.

## Abbreviations

AHRQ: Agency for Healthcare Research and Quality
BRFSS: Behavioral Risk Factor Surveillance System
CMS: Centers for Medicare and Medicaid Services
COE: centers of excellence
DOI: diffusion of innovation
HCUP: Healthcare Cost and Utilization Project
NCD: National Coverage Decision
PC: percent change
SID: State Inpatient Database
